# Association between Neovascular Age-Related Macular Degeneration and Dementia: A Population-Based Case-Control Study in Taiwan

**DOI:** 10.1371/journal.pone.0120003

**Published:** 2015-03-06

**Authors:** Shiu-Dong Chung, Cha-Ze Lee, Li-Ting Kao, Herng-Ching Lin, Ming-Chieh Tsai, Jau-Jiuan Sheu

**Affiliations:** 1 Department of Ophthalmology, Taipei Medical University Hospital, Taipei, Taiwan; 2 Sleep Research Center, Taipei Medical University Hospital, Taipei, Taiwan; 3 Department of Internal Medicine, National Taiwan University Hospital, Taipei, Taiwan; 4 Graduate Institute of Life Science, National Defense Medical Center, Taipei, Taiwan; 5 School of Health Care Administration, Taipei Medical University, Taipei, Taiwan; 6 Division of Gastroenterology, Department of Internal Medicine, Cathay General Hospital, Taipei, Taiwan; 7 Department of Neurology, Taipei Medical University Hospital, Taipei, Taiwan; 8 Department of Neurology, School of Medicine, College of Medicine, Taipei Medical University, Taipei, Taiwan; Radboud University Nijmegen Medical Centre, NETHERLANDS

## Abstract

**Background:**

Most available studies focusing on the association between neovascular age-related macular degeneration (AMD) and dementia have conflicting results. This study aimed to investigate the association between previously diagnosed AMD and dementia using a population-based dataset in Taiwan.

**Methods:**

Data for this case-control study were retrospectively collected from the Taiwan National Health Insurance Research Database. We identified 13,402 subjects who had a diagnosis of dementia as cases, and 40,206 subjects without dementia as controls. A conditional logistic regression was used to examine the association of dementia with previously diagnosed neovascular AMD.

**Results:**

We found that of the study sample of 53,608 subjects, 1.01% had previously diagnosed neovascular AMD, 1.35% and 0.90% for cases and the controls, respectively (p<0.001). The conditional logistic regression analysis suggested that the odds ratio of prior neovascular AMD for cases was 1.37 (95% confidence interval: 1.14~1.65) compared to the controls after adjusting for subjects’ age, monthly income, geographic location, urbanization level, and hyperlipidemia, diabetes, hypertension, stroke, ischemic heart disease, and whether or not a subjects underwent cataract surgery prior to index date than controls.

**Conclusions:**

Dementia subjects were associated with a higher proportion of prior neovascular AMD than were the controls.

## Introduction

Along with increasing numbers of elderly people, dementia has become an emerging public health challenge around the world. There were an estimated 35.6 million people afflicted by dementia in 2010 worldwide, and as the world population ages, this number will greatly increase in the future [1]. Dementia can be caused by degenerative, vascular, or other non-degenerative etiologies, and aging is recognized as one of the major risk factors for degenerative and vascular dementias [2]. As no specific underlying cause has been identified, Alzheimer's disease (AD), the most common form of dementia, is considered to be an age-related neurodegenerative disease that evolves from complex interactions among genetic susceptibility, aging, and other influential factors which trigger neurodegeneration in the brain [3,4].

Age-related macular degeneration (AMD) is the leading cause of visual impairment and loss in aged people in Western countries [5]. AMD was also found to be a common eye disorder in elderly ethnic Chinese people in Taiwan, and the prevalence rate of AMD was similar to those of other ethnic groups [6,7]. AMD is initially characterized by drusen accumulation in the central retina and progresses with choroidal neovascularization or geographic atrophy of the retina in the advanced stage [8]. The pathogenesis of AMD is not fully known and is highly complicated with multi-factorial interactions of aging, genetic, and environmental risk factors [9]. The central component of the pathophysiology of AMD is degeneration and dysfunction of retinal pigment epithelial cells and perturbation of the physiological integrity of neighboring photoreceptor neural cells, i.e., rods and cones. Therefore, AMD is regarded as a neurodegenerative disease of the eye [10,11].

A body of molecular studies suggested that chronic oxidative stress and neuroinflammation, derangement of the processing and degradation of dysfunctional cellular components, and alterations of neuronal homeostasis are common biological pathomechanisms of age-associated neurodegenerative diseases including AMD and AD [4,10,11]. However, most available data focusing on the association between AMD and dementia are from studies with a cross-sectional design and the results remain conflicting [12–17]. In addition, population-based studies in Asian populations are few and have not shown any significant associations [16], and, to the best of our knowledge, large epidemiologic investigations regarding the association between AMD and dementia are still lacking in Chinese populations. Although it only represents 10%~15% of total AMD cases, neovascular AMD, one of the major types of advanced AMD, accounts for most cases of serious visual impairment or blindness due to AMD [8]. Therefore, the aim of this study was to explore the association between neovascular AMD and dementia in a Chinese population using a large, population-based dataset in Taiwan.

## Methods

### Database

This case-control study is based on a retrospective analysis of administrative claims data taken from the Longitudinal Health Insurance Database 2000 (LHID2000). Taiwan began its National Health Insurance (NHI) program in 1995 to provide affordable and easily accessible medical care for all its citizens (with a copayment of only around US$3~5 per outpatient visit). The LHID2000 consists of claims data and registration files of 1,000,000 individuals randomly sampled from the 2000 Registry for Beneficiaries (*n* = 23.72 million) of the Taiwan NHI program. Many researchers as well as the Taiwan National Health Research Institute have demonstrated the high validity of data derived from the Taiwanese NHI program [18,19]. The LHID2000, which was open to the researchers in Taiwan, was available from the Taiwan National Health Research Institute (http://nhird.nhri.org.tw/date_01.html).

This study was approved by institutional review board (IRB) of Taipei Medical University's IRB (TMU-JIRB 201403011).

### Selection of Cases and Controls

To select cases for this study, we first identified 14,642 subjects from the LHID2000 who had received a diagnosis of dementia (ICD-9-CM codes 290.0~290.4, 294.1, 331.0~331.2, or 331.82) during ambulatory care visits between January 2002 and December 2011. Since administrative datasets are always criticized for their diagnostic validity, this study only included those subjects who had been diagnosed with dementia at least twice during the period between 2002 and 2011, with at least one diagnosis being made by a certified neurologist. We indicated their first dementia diagnosis as the index date in this study. We excluded patients under 40 years of age because of the very low prevalence of dementia in that age group (*n* = 154). We further excluded those who had a history of major psychosis or a substance-related disorder (ICD-9-CM codes 291~299 or 303~305) prior to the index date (*n* = 1086). Ultimately, 13,402 subjects with dementia were selected as cases in this study.

We retrieved three matched controls (*n* = 40,206) per case from the remaining beneficiaries of the LHID2000. Controls were matched by gender, age group (40~49, 50~59, 60~69, 70~79, and >79 years), and index year. While for cases, the year of the index date was the year in which the cases received their first dementia diagnosis, for controls, the year of the index date was simply a matched year in which the controls had a medical utilization. We further assigned the date of their first use of ambulatory care occurring during that matched year as the index date for the controls. In addition, we assured that none of the selected controls had received a dementia diagnosis since initiation of the Taiwan NHI program in 1995. We also assured that none of the selected controls had received a diagnosis of major psychosis or a substance-related disorder prior to the index date.

In this study, we calculated the odds of having previously been diagnosed with neovascular AMD between cases and controls. We identified cases with neovascular AMD based on ICD-9-CM codes 362.42, 362.43, 362.52, or 362.53. In order to increase the diagnostic validity, this study only included subjects who had received two or more diagnoses of neovascular AMD prior to the index date.

### Statistical Analysis

All analyses were conducted using the SAS system (SAS System for Windows, vers. 8.2, SAS Institute, Cary, NC). We used a Chi-square test to compare differences in monthly income (NT$0~15,840, 15,841~25,000, ≥25,001) (In 2011, the average exchange rate was US$1 ≈ NT$29), geographic location (northern, central, eastern, and southern Taiwan), and urbanization level of the patient’s residence (5 levels, with 1 being the most urbanized and 5 being the least) between cases and controls. We also took medical comorbidities including hyperlipidemia, diabetes, hypertension, stroke, and ischemic heart disease into consideration in this study. These selected medical comorbidities are all potential risk factors for dementia, and they were only included if they were diagnosed before the index date. In addition, we took whether or not a subject underwent cataract surgery prior to index date into consideration in the regression model. A conditional logistic regression (conditioned on gender and index year) was used to examine the association of dementia with previously diagnosed neovascular AMD. The conventional *p*≤0.05 was used to assess statistical significance.

## Results

Of the 53,608 sampled subjects, the mean age was 76.1 years with a standard deviation of 9.9 years; the mean ages for cases and controls were 76.3 and 76.0 years, respectively (*p* = 0.721). Table 1 presents the demographic characteristics and medical comorbidities of cases and controls. After matching for gender and age group, cases had a higher prevalence of previous comorbidities including hyperlipidemia (10.6% vs. 8.1%, *p*<0.001), diabetes (20.8% vs. 19.5%, *p* = 0.001), hypertension (49.1% vs. 43.3%, *p*<0.001), ischemic heart disease (12.7% vs. 10.2%, *p*<0.001), and stroke (27.6% vs. 14.5%, *p*<0.001) than controls. In addition, cases were more likely to have monthly incomes of <NT$15,841 (*p*<0.001) than controls. There was no significant difference in geographic region or urbanization level between cases and controls.

**Table 1:** Demographic characteristics of subjects with dementia and controls in Taiwan (n = 53,608).

Variable	Patients with dementia (*n* = 13,402)		Controls (*n* = 40,206)		*p* value
	Total no.	%	Total no.	%	
Age (years)					>0.999
40∼49	180	1.3	540	1.3	
50∼59	533	4.0	1599	4.0	
60∼69	1698	12.7	5094	12.7	
70∼79	5039	37.6	15,117	37.6	
80∼89	5200	38.8	15,600	38.8	
>90	752	5.6	2256	5.6	
Gender					>0.999
Male	6344	47.3	19,032	47.3	
Female	7058	52.7	21,174	52.7	
Monthly Income					<0.001
<NT$1∼15,841	8800	65.7	22,504	56.0	
NT$15,841∼25,000	4164	31.0	15,059	37.4	
≥NT$25,001	438	3.3	2643	6.6	
Hyperlipidemia	1421	10.6	3257	8.1	< 0.001
Diabetes	2789	20.8	7840	19.5	0.001
Hypertension	6580	49.1	8860	43.3	< 0.001
Stroke	3698	27.6	5836	14.5	< 0.001
Ischemic heart disease	1702	12.7	4101	10.2	< 0.001
Prior cataract surgery	218	1.6	843	2.1	< 0.001
Geographic region					0.113
Northern	5761	43.0	17,335	43.1	
Central	3317	24.7	10,071	25.0	
Eastern	3886	29.0	11,649	29.0	
Southern	438	3.3	1151	2.9	
Urbanization level					0.264
1 (most urbanized)	3372	25.2	10,320	25.7	
2	3510	26.1	10,330	25.7	
3	1873	14.0	5788	14.4	
4	2374	17.7	6892	17.1	
5 (least urbanized)	2273	17.0	6876	17.1	

Table 2 presents the prevalence of prior neovascular AMD between cases and controls. It reveals that 542 (1.01%) of sampled subjects had neovascular AMD before the index date; neovascular AMD was found in 181 (1.35%, 95% CI = 1.17%-1.56%) cases and in 361 (0.90%, 95% CI = 0.81%-0.99%) controls (*p*<0.001). Correspondingly, the conditional logistic regression analysis suggested that the odds ratio (OR) of prior neovascular AMD for cases was 1.51 (95% confidence interval (CI): 1.26∼1.81) compared to the controls.

**Table 2:** Prevalence, crude odds ratios (ORs), and 95% confidence intervals (CIs) for neovascular age-related macular degeneration among sampled subjects.

Presence of neovascular age-related macular degeneration	Total (*n* = 53,608)		Subjects with dementia (*n* = 13,402)		Controls (*n* = 40,206)	
	*n*, %		*n*, %		*n*, %	
Yes	542	1.01	181	1.35	361	0.90
No	53,066	98.99	13,221	98.65	39,845	99.10
OR (95% CI)	—		1.51*** (1.26∼1.81)		1.00	

Notes: The OR was calculated by a conditional logistic regression which was conditioned on gender, age group, and the year of the index date.

*** *p*<0.001.

Table 3 further indicates that after adjusting for subjects’ age, monthly income, geographic location, urbanization level, and hyperlipidemia, diabetes, hypertension, stroke, ischemic heart disease, and whether or not a subjects underwent cataract surgery prior to index date, subjects with dementia were more likely than controls to have been diagnosed with neovascular AMD before the index date (OR: 1.37; 95% CI: 1.14∼1.65; *p*<0.001). As expected, the medical comorbidities of hyperlipidemia, hypertension, and stroke were significantly associated with dementia.

**Table 3:** Covariate-adjusted odds ratios (ORs) and 95% confidence intervals (CIs) for neovascular age-related macular degeneration among sampled subjects (n = 18,424).

Variable	Presence of dementia		
	Adjusted OR	95% CI	*p* value
Prior neovascular age-related macular degeneration			
Yes	1.37	1.14∼1.65	<0.001
No (reference group)	1.00		
Age	1.01	1.01–1.02	<0.001
Monthly income			
<NT$15,841 (reference group)	1.00		
NT$15,841∼25,000	0.67	0.63∼0.70	<0.001
≥NT$25,001	0.50	0.45∼0.56	<0.001
Hyperlipidemia	1.25	1.16∼1.34	<0.001
Diabetes	1.04	0.99∼1.10	0.105
Hypertension	1.41	1.36∼1.47	<0.001
Ischemic heart disease	0.97	0.91∼1.03	0.359
Stroke	2.21	2.11∼2.32	<0.001
Prior cataract surgery	0.73	0.63–0.85	<0.001
Geographic region			
Northern (reference group)	1.00		
Central	1.06	0.99∼1.12	0.057
Eastern	1.09	1.04∼1.15	<0.001
Southern	1.16	1.03∼1.30	0.018
Urbanization level			
1 (reference group)	1.00		
2	1.05	0.99∼1.11	0.099
3	1.04	0.98∼1.12	0.217
4	1.19	1.11∼1.28	<0.001
5	1.21	1.13∼1.31	<0.001

Note: all variables listed in the table were adjusted for in the same logistic regression model.

In addition, according to a stepwise multiple logistic regression model (Table 4), the variables including stroke (*p*<0.001), hypertension (*p*<0.001), monthly income (*p*<0.001), age (*p*<0.001), hyperlipidemia (*p*<0.001), urbanization level (*p*<0.001), prior cataract surgery (*p*<0.001), age-related macular degeneration (*p*<0.001) and geographic location (*p*<0.05) were independently associated with dementia.

**Table 4:** Model of variables independently associated with dementia revealed by a stepwise multiple logistic regression.

Variable	OR	95% CI	*p* value
Stroke	2.22	2.12∼2.33	<0.001
Hypertension	1.41	1.36∼1.47	<0.001
Monthly income			
≥NT$25,001	0.50	0.45∼0.56	<0.001
NT$15,841∼25,000	0.67	0.64∼0.70	<0.001
Age	1.01	1.01∼1.01	<0.001
Hyperlipidemia	1.25	1.16∼1.34	<0.001
Urbanization level			
5	1.18	1.11∼1.25	<0.001
4	1.15	1.09∼1.22	<0.001
Prior cataract surgery	0.73	0.63∼0.85	<0.001
Age-related macular degeneration	1.36	1.13∼1.64	<0.001
Geographic region			
Southern	1.10	1.05∼1.16	0.005
Eastern	1.17	1.04∼1.32	0.033
Central	1.07	1.01∼1.27	0.018

## Discussion

The previous population-based cross-sectional epidemiologic studies examining the relationship between AMD and dementia or cognitive decline showed inconsistent results [12–17]. The Atherosclerosis Risk in Communities Study and the Cardiovascular Health Study suggested an association between low cognitive function and early AMD [12,15]. In addition, the Age-Related Eye Disease Study, the Blue Mountains Eye Study, and the Tromsø Eye Study all have shown that advanced AMD is associated with cognitive impairment [13,14,17]. To the contrary, the Singapore Malay Eye Study could not document any significant associations between AMD and cognitive dysfunction [16], and dementia and AD were not found to be associated with early AMD in the Cardiovascular Health study [15]. These inconsistencies may have been due to differences in methodology and design and racial differences in the prevalences of AMD and cognitive impairment. The reasons why those studies could not provide evidence of an association between advanced AMD and cognitive dysfunction may be related to the small number of advanced AMD cases in the study populations and insufficient statistical power [12,15,16]. In addition, cross-sectional studies cannot yield information regarding causal relationships, whereas case-control studies are more useful for generating hypotheses from relatively rare diseases. In view of these disadvantages, the present nationally representative case-control study was more appropriate in that we enroll a sufficient number of samples to provide adequate statistical power and was therefore suitable for assessing the association between advanced AMD and dementia.

In our study among Taiwanese (ethnic Chinese) aged ≥40 years, a significantly higher proportion of prior neovascular AMD was found among subjects with dementia compared to controls after adjusting for sociodemographic characteristics and comorbid medical disorders including cardiovascular risk factors, ischemic heart disease, and stroke. Our findings are in agreement with the findings of the Rotterdam Study, which support the potential association between AMD and dementia [20]. There are lines of evidence to suggest that AD and vascular dementia are associated with vascular risk factors, including hypertension, diabetes, and hyperlipidemia which are also well acknowledged to be risk factors for neovascular AMD [21–24]. Thus, as in the Rotterdam Study, a possible explanation for the association between neovascular AMD and dementia is a result of the effects of vascular risk factors. Whereas our analysis included adjustments for hypertension, diabetes, and hyperlipidemia, our results suggest that vascular risk factors are not the sole mechanism contributing to the association between neovascular AMD and dementia.

The actual mechanisms contributing to the association between neovascular AMD and dementia are unclear. The pathological hallmarks of AMD and AD share several similar features. Neurotoxic amyloid β is present in amyloid plaques of AD brains and also in drusens of AMD retinas [10,11]. Molecular constituents including inflammatory mediators, elements of ubiquitin-proteasome and autophagy-lysosomal systems, and complement components were detected in both drusens and amyloid plaque [10,11]. As retinal pigment epithelial cells senesce and their capacity to maintain the integrity of the function of the retina declines in the elderly, the generation of reactive oxygen species (ROS) and oxidative stress increase in the retina [25]. Mounting evidence suggests that complicated interactions between excessive ROS, oxidative stress, and mitochondrial and lysosomal dysfunctions also contribute to the development and progression of AD [26]. Another possible mechanism that links neovascular AMD to dementia lies in serious visual impairment and blindness caused by advanced AMD. Serious visual impairment or blindness, deprivation of an organ essential for sensory functioning, may limit patients' activities of daily living, hinder participation in physical and mental leisure activities which are healthy to the brain, lead to an attenuation in the complexity of neuronal synapses and reduced cognitive reserve, and ultimately result in aggravation of cognitive impairment in the elderly [27,28]. In addition, elderly people with severe visual impairment due to advanced AMD suffer from considerable psychological distress and depression, which are potentially associated with increased risk of cognitive decline and dementia [29,30].

A particular strength of this study is the use of a nationwide population-based dataset that provides a sufficient sample size and statistical power to explore the association between neovascular AMD and dementia. Nevertheless, some limitations to our study should be addressed. First, neovascular AMD and dementia diagnoses, which rely on administrative claims data and International Classification of Diseases codes, may be less precise than those made according to standardized criteria. This is a major limitation of this study compared with previous studies that used standardized diagnostic examinations of patients. However, to preclude miscoding or inaccurate medical claims and to ensure diagnostic validity, the NHI Bureau of Taiwan maintains a regular cross-checking system with assessment and scrutiny of chart records from every hospital, followed by heavy penalties if discrepancies or instances of malpractice are discovered. Moreover, previous studies that used the NHI Research Database demonstrated that it is of acceptable quality to provide reasonable estimates for epidemiological studies of neovascular AMD and dementia [31,32]. To avoid misclassification and increase the AMD diagnostic accuracy, we did not include early AMD patients in our study because early AMD patients are usually asymptomatic and tend to be more inconsistently classified by ophthalmologists [33]. On the contrary, we limited our study sample to neovascular AMD patients because neovascular AMD usually manifests with rapid worsening of vision and its fundoscopic signs, including exudates, hemorrhage, and retinal detachment, are characteristic of the disease and allow different ophthalmologists to make straightforward, accurate diagnoses.

Second, there may be a surveillance bias because dementia patients are more likely to have frequent evaluations and thus more AMD would be detected, and vice versa. However, clinical evidence linking dementia and AMD is uncertain and not conclusive. In clinical practice, dementia patients are usually followed-up by their neurologists and AMD patients by ophthalmologists. Dementia patients visit an ophthalmologist only when they have visual symptoms and then receive fundoscopic examination to diagnose AMD. Likewise, AMD patients visit a neurologist only when they have cognitive or behavioral symptoms and receive neuropsychological testing and brain imaging to diagnose dementia.

Third, the administrative dataset did not provide individual information on cigarette smoking number and years which might potentially modify the relationship between neovascular AMD and dementia. From the findings of the Rotterdam Study, the association between AMD and dementia may be partially explained by the effect of smoking [20]. Therefore, smoking can lead to AMD and might bias the reported association of AMD with cognitive loss.

Fourth, treatment of AMD such as anti-vascular endothelial growth factor (anti-VEGF) agents may have a confounding effect on the association between AMD and dementia. However, because we identified cases from the LHID2000 between January 2002 and December 2011, and Taiwan’s NHI has approved the use of anti-VEGF agents for treatment of AMD since 2011, the effect of anti-VEGF medications does not seem significant in this study.

Fifth, the study population mainly consisted of ethnic Chinese, and therefore the ability to generalize the results to other ethnic populations is uncertain. Finally, the dataset only allowed us to trace the medical utilization of sampled patients back to 1996. Therefore, we could not rule out those cases and controls who had neovascular AMD or dementia prior to 1996, and this could have compromised our findings.

Despite these limitations, our population-based study found that dementia patients were associated with a higher proportion of prior neovascular AMD than control among Taiwanese (ethnic Chinese) and supported the association between neovascular AMD and dementia. Further studies are needed to confirm the association found in the present study and to clarify the underlying pathophysiological mechanisms.
